# A Randomized Evaluation of MoodFX, a Patient-Centred e-Health Tool to Support Outcome Measurement for Depression: Une évaluation randomisée de MoodFX, un outil de santé en ligne centré sur le patient pour soutenir la mesure du résultat dans la dépression.

**DOI:** 10.1177/07067437241245331

**Published:** 2024-04-11

**Authors:** Victor W. Li, Jaspreet Sahota, Deea K. Dev, Dib D. Gill, Vanessa C. Evans, Auby Axler, Trisha Chakrabarty, André Do, Kamyar Keramatian, John-Jose Nunez, Edwin M. Tam, Lakshmi N. Yatham, Erin E. Michalak, Jill K. Murphy, Raymond W. Lam

**Affiliations:** 1 Department of Psychiatry, 8166 University of British Columbia, Vancouver, Canada; 2 Department of Psychiatry, Université de Montréal, Montreal, Canada

**Keywords:** depressive disorders, antidepressants, e-mental health, randomized controlled trial, troubles dépressifs, antidépresseurs, santé mentale en ligne, essai randomisé contrôlé

## Abstract

**Background:**

e-Health tools using validated questionnaires to assess outcomes may facilitate measurement-based care for psychiatric disorders. MoodFX was created as a free online symptom tracker to support patients for outcome measurement in their depression treatment. We conducted a pilot randomized evaluation to examine its usability, and clinical utility.

**Methods:**

Patients presenting with a major depressive episode (within a major depressive or bipolar disorder) were randomly assigned to receive either MoodFX or a health information website as the intervention and control condition, respectively, with follow-up assessment surveys conducted online at baseline, 8 weeks and 6 months. The primary usability outcomes included the percentage of patients with self-reported use of MoodFX 3 or more times during follow up (indicating minimally adequate usage) and usability measures based on the System Usability Scale (SUS). Secondary clinical outcomes included the Quick Inventory of Depressive Symptomatology, Self-Rated (QIDS-SR) and Patient Health Questionnaire (PHQ-9).

**Results:**

Forty-nine participants were randomized (24 to MoodFX and 25 to the control condition). Of the 23 participants randomized to MoodFX who completed the user survey, 18 (78%) used MoodFX 3 or more times over the 6 months of the study. The mean SUS score of 72.7 (65th–69th percentile) represents good usability. Compared to the control group, the MoodFX group had significantly better improvement on QIDS-SR and PHQ-9 scores, with large effect sizes and higher response rates at 6 months. There were no differences between conditions on other secondary outcomes such as functioning and quality of life.

**Conclusion:**

MoodFX demonstrated good usability and was associated with reduction in depressive symptoms. This pilot study supports the use of digital tools in depression treatment.

## Plain Language Summary

E-health tools may be useful for measuring and tracking symptoms and other outcomes during treatment for depression. This study is a randomized evaluation of MoodFX, a free web-based app that helps patients track their symptoms using validated questionnaires, and also offers depression information and self-management tips. A total of 49 participants with clinical depression were randomized to using MoodFX or a health information website, for 6 months. In a survey, the participants that used MoodFX found it easy and useful to use. In addition, the participants that used MoodFX had greater improvement in depressive symptoms after 6 months, compared to those who used the health information website. These results suggest that MoodFX may be a useful tool to monitor outcomes and support depression treatment.

## Abstract (fr)

**Contexte:**

Les outils de santé en ligne qui utilisent des questionnaires validés pour évaluer les résultats peuvent faciliter les soins basés sur la mesure (SBM) pour les troubles psychiatriques. MoodFX a été créé à titre de suivi des symptômes en ligne gratuit pour soutenir la mesure des résultats des patients pour leur traitement de la dépression. Nous avons mené une évaluation randomisée pilote pour en examiner l’utilisabilité et l’utilité clinique.

**Méthodes:**

Les patients présentant un épisode dépressif majeur (dans le cadre d’un trouble dépressif majeur ou d’un trouble bipolaire) ont été assignés au hasard à recevoir soit un MoodFX ou un site Web d’information de santé comme condition d’intervention et de contrôle, respectivement, avec des sondages d’évaluation de suivi menés en ligne à la base, après 8 semaines et 6 mois. Les premiers résultats d’utilisabilité comportaient le pourcentage de patients ayant un usage auto-déclaré de MoodFix trois fois ou plus durant le suivi (indiquant un usage minimalement adéquat) et des mesures d’utilisabilité basées sur l’Échelle d’utilisabilité du système (EUS). Les résultats cliniques secondaires comprenaient l’Inventaire rapide de la symptomatologie dépressive auto-déclarée (QIDS-SR) et le questionnaire de santé du patient (PHQ-9).

**Résultats:**

Quarante-neuf participants ont été randomisés (24 au MoodFX et 25 à la condition de contrôle). Sur les 23 participants randomisés au MoodFX qui ont terminé le sondage de l’utilisateur, 18 (78%) ont utilisé MoodFX trois fois ou plus au cours des six mois de l’étude. Le score EUS moyen de 72,7 (65–69^e^ percentile) représente une bonne utilisabilité. Comparé au groupe témoin, le groupe MoodFX avait une amélioration significativement meilleure aux scores QIDS-SR et PHQ-9 avec de grandes tailles d’effet et plus de taux de réponses à 6 mois. Il n’y avait pas de différences entre les conditions aux autres résultats secondaires comme le fonctionnement et la qualité de vie.

**Conclusion:**

MoodFX a démontré une bonne utilisabilité et était associé à la réduction des symptômes dépressifs. Cette étude pilote soutient l’usage des outils numériques dans le traitement de la dépression.

## Introduction

Depression is a common mental health condition that affects more than 280 million people worldwide, and the number is increasing every year.^1^ Depression is a complex condition characterized by a wide range of symptoms, including sadness, loss of interest, fatigue, cognitive dysfunction and suicidal thoughts, and can be associated with significant functional impairment and poor quality of life.^2^ Despite the availability of evidence-based treatment options, including pharmacotherapy and psychotherapy, a significant number of patients with depression do not receive adequate care and experience poor outcomes.^3^

Measurement-based care (MBC) has emerged as an effective way to improve the quality of care for depression. MBC involves tracking symptoms over time using standardized measures to guide pharmacological and non-pharmacological treatment decisions.^4^ For pharmacological decision making, MBC can help to assess medication response and the need for dose adjustment or drug switching. It can also help guide non-pharmacological approaches such as psychotherapy to better assess clinical response or non-response. However, despite evidence demonstrating improved outcomes,^5,6^ MBC is still not routinely used by clinicians.^7^ Barriers to the use of MBC include a lack of knowledge of which scales to use and how to incorporate measurements into clinical charting systems, and the (mis)perception that extra time is needed for repeated assessments that takes away from clinical encounter time.^8,9^

Given the burgeoning e-Health and digital solutions for mental health since the COVID-19 pandemic,^10^ online and mobile mood tracking apps may address some of these challenges to outcome measurement, a core component of MBC. Although there are many benefits of outcome measurement for patients with depression, there are also potential harms from using symptom trackers. Users may become frustrated with repeatedly completing the same questionnaires, or with lack of improvement in scores despite treatment. Patients with depression often experience ruminations and worry, and high scores on questionnaires may exacerbate these depressive symptoms. Hence, it is important to evaluate the user experience for symptom tracking tools.

MoodFX (pronounced “mood effects”) is an online patient-centered e-Health tool that was created by a team (which included Raymond Lam, Vanessa Evans, Erin Michalak and Lakshmi Yatham) at the University of British Columbia (UBC) to support MBC for depression. Optimized for use on mobile devices, this free user-friendly web app allows users to track their symptoms and functional outcomes using standardized, validated questionnaires and provides real-time feedback. Results can then be displayed or printed to share with clinicians to support MBC. MoodFX includes a reminder feature so that users can receive regular reminders via text or email to complete the assessment, such as entering their next clinician appointment date to receive a reminder to complete the questionnaires the day before their appointment. Finally, MoodFX users can opt in to receive weekly self-management tips by text or email.

Given that there are few rigorous studies examining e-Health tools to support outcome measurement, we conducted a randomized evaluation study of MoodFX. The purpose of this pilot study was to evaluate the usability of MoodFX and obtain exploratory data for its effectiveness in improving outcomes for patients with depression. Our primary hypothesis was that MoodFX would be acceptable to patients with depression and easy to use. Secondary hypotheses included that the use of MoodFX would result in greater reduction in depressive and anxiety symptoms and improve functionality, compared to a control condition.

## Methods

### Setting and Participants

This trial was registered at clinicaltrials.gov (NCT 03762460) and approved by the UBC Clinical Research Ethics Board (H17-02786-A009). The study was conducted at the UBC Mood Disorders Centre, an outpatient clinic that provides consultation assessments on referral by family physicians and psychiatrists. Patients are assessed by psychiatrists with mood disorders expertise and treatment recommendations are given back to the referral source. Consecutive patients meeting eligibility criteria were approached to participate. Inclusion criteria were: (1) age 19 to 65 and capable of informed consent by the opinion of the clinic psychiatrist; (2) a diagnosis of major depressive episode by DSM-5 criteria as determined by the clinic psychiatrist; (3) at least moderate severity as defined by a score of 10 or higher on the Quick Inventory of Depressive Symptomatology, Self-Rated (QIDS-SR)^11^; (4) access to an internet-enabled computer or mobile device and (5) ability to read and understand English or French, since MoodFX is currently available in those languages only. Exclusion criteria were (1) active psychotic or substance use disorder and (2) severe suicidality as judged by the clinic psychiatrist.

### Study Procedures

As the study was conducted during the COVID pandemic, patients referred to the clinic were assessed virtually via Zoom and the study was conducted remotely to ensure social distancing. Eligible participants who provided written informed consent were randomly assigned 1:1 to either (1) MoodFX or (2) a mental health information internet site (www.heretohelp.ca). At the baseline visit, study staff helped participants access and set up the MoodFX or heretohelp.ca website on their mobile device or home computer. Subsequently, patients returned to their usual care providers with a letter explaining the study and how these interventions might support their practice. No additional efforts were made to enforce or verify that MoodFX or the control web site would be used by the participants and/or providers.

### Interventions

The active intervention, MoodFX, includes several features to support depression self-management and facilitate MBC (Table 1). The validated symptom and outcome scales are meant to be completed by the patient regularly during treatment, with results shared with their clinician to support clinical decision making. Users can set reminders (by email or SMS) to complete the scales on a weekly, bi-weekly or monthly basis. They can also receive alerts to complete the scales prior to their clinician appointments, with the aim of providing their clinician with the most up-to-date results. The scale results can be printed, emailed or shown to clinicians on a mobile device during their appointment. In addition to outcome tracking, MoodFX provides patients with resources and psychoeducation both through the “FAQ” page and the “Resources” page. The “Resources” section provides additional online resources for psychoeducation and web-based psychotherapy. Both sections are designed to provide patients with knowledge and tools regarding their diagnosis so that they can better manage their symptoms at work and at home. MoodFX complies with Canadian data privacy protection laws and regulations. All usage data are stored on a secure UBC server and managed in compliance with the BC Freedom of Information and Protection of Privacy Act. Unfortunately, for technical reasons we were unable to obtain the back-end usage data for analysis.

**Table 1.** Components of MoodFX.

Feature	Comment	Rationale
***Symptom assessment:***		
Personal Health Questionnaire (PHQ-9)^12^	Commonly used 9-item scale for depression symptoms	Symptom scores are recorded and trended over time. The data can be valuable as feedback for patients and for providers in guiding clinical decision making
Generalized Anxiety Disorder scale (GAD-7)^13^	Commonly used 7-item scale for generalized anxiety symptoms	
Perceived Deficits Questionnaire, 5-item (PDQ-5)^14^	5-Item scale assessing frequency of problems with memory, concentration, organization	
***Functioning assessment:***		
Sheehan Disability Scale (SDS)^17^	Functional impairment scale for the domains of work/school, family life and social life	Functional improvement does not always correlate with symptom improvement, requiring it to be measured separately
Lam Employment Absence and Productivity Scale (LEAPS)^16^	Functional impairment scale for work functioning and productivity	
***Side effect assessment:***		
Frequency, Intensity and Burden of Side Effects Ratings (FIBSER)^15^	Scale assessing severity, onset and frequency of side effects	Side effects affect treatment compliance, but symptoms can also be non-specific. Detailed tracking can be helpful for treatment decisions
***Psychoeducation:***		
Frequently Asked Questions	Explains common questions about depressive symptoms, mood tracking and using measurement-based care with physicians	MoodFX provides a limited selection of additional relevant resources and information for patients
Self-Management Tips	Optional email or SMS subscription to receive weekly/monthly tips on self-management	
Resources	Links to online websites with patient-oriented psychoeducation	

The control condition was a website, https://www.heretohelp.bc.ca/, which provides health information about depression and other mental health conditions but has no active symptom tracking capability. To maintain blinding and minimize contamination between the 2 conditions, participants were informed that the study objective was to examine the use of web-based e-Health sites without specifically mentioning MBC or MoodFX.

No specific clinical interventions were used in this study. Patients received a one-time assessment by the clinic psychiatrist and no further clinic follow up was offered. A consultation report with treatment recommendations was sent back to the referring clinician and patients returned to them for ongoing usual care.

### Assessments

At initial assessment, clinical data including demographics, other psychiatric and nonpsychiatric medical diagnoses, number and duration of previous depressive episodes, severity and duration of current depressive episode, past hospitalizations and treatments, and current and concomitant medications were collected. Participants completed self-rated assessments on a secure online site at baseline, 8 weeks and 6 months. These assessments included: QIDS-SR and Patient Health Questionnaire-9 (PHQ-9)^12^ for depressive symptoms; Generalized Anxiety Disorder-7 Scale (GAD-7)^13^ for anxiety; Perceived Deficits Questionnaire – Depression, 5-item (PDQ-5)^14^ for cognitive difficulties; Frequency, Intensity, and Burden of Side Effects Ratings (FIBSER)^15^ for medication side effects; Lam Employment Absence and Productivity Scale (LEAPS)^16^ for work functioning; Sheehan Disability Scale (SDS)^17^ for functional impairment; Quality of Life Satisfaction and Enjoyment Questionnaire Scale (QLESQ)^18^ for quality of life; EuroQol Group: 5 Dimension, 5-Level (EQ-5D-5L)^19^ for self-perceived health status; and a brief Health Economics Assessment (HEA) for healthcare service utilization.

At 6 months, participants assigned to MoodFX completed a user survey that included the System Usability Scale (SUS),^20^ a scale originally developed in systems engineering to capture user sentiment about an interactive system, but now widely used to evaluate online and digital programmes. The SUS is a short questionnaire composed of 10 items related to the ease or difficulty of learning and using a system, such as “I think I would like to use the tool frequently” and “I thought the tool was easy to use.” Responses to the statements were based on a 5-point Likert scale with responses of Strongly Agree, Agree, Neutral, Disagree, and Strongly Disagree. Responses were assigned scores of 0 to 4, with higher scores indicating better usability, summed for the 10 questions and then multiplied by 2.5 for a final score. The common benchmark score of 68 corresponds to 50th percentile performance.^21^ A final question on the user survey asked, “Over the last 6 months, how many times did you use the tool?” with responses of Never, Once, Twice, and Three or More Times. The last response was considered a minimally adequate usage of outcome measurement for MBC, for example, obtaining symptom scores at baseline and 2 follow-up time points.

### Statistical Analysis and Sample Size

The sample size was determined by comparison to other pilot usability studies. For the secondary outcomes, this sample size allows detection of large effect sizes. The primary usability outcome was the percentage of participants who reported minimally adequate usage of MoodFX, defined as 3 or more uses over the 6-month study period as captured by the user survey. The secondary usability outcome was ease of use, as assessed by the SUS score included in the same survey. Secondary clinical outcomes include changes in validated clinical measures (symptoms, functioning and quality of life) from baseline to follow up at 8 weeks and 6 months.

Missing data were imputed with last observation carried forward as a conservative estimate for true effect sizes. Standard parametric (mixed model analysis of variance (ANOVA) and T-Test) and non-parametric (Fisher's exact test) analyses were used as appropriate. Effect sizes were calculated using Cohen's d statistic. Clinical outcome scores over time, percentage of responders (≥50% improvement in scores from baseline to follow up), and percentage of participants no longer meeting the threshold QIDS-SR criterion at baseline (i.e., QIDS-SR < 10) were examined. Because of the exploratory nature of these secondary analyses, no corrections for multiple comparisons were used.

## Results

**Figure 1.**
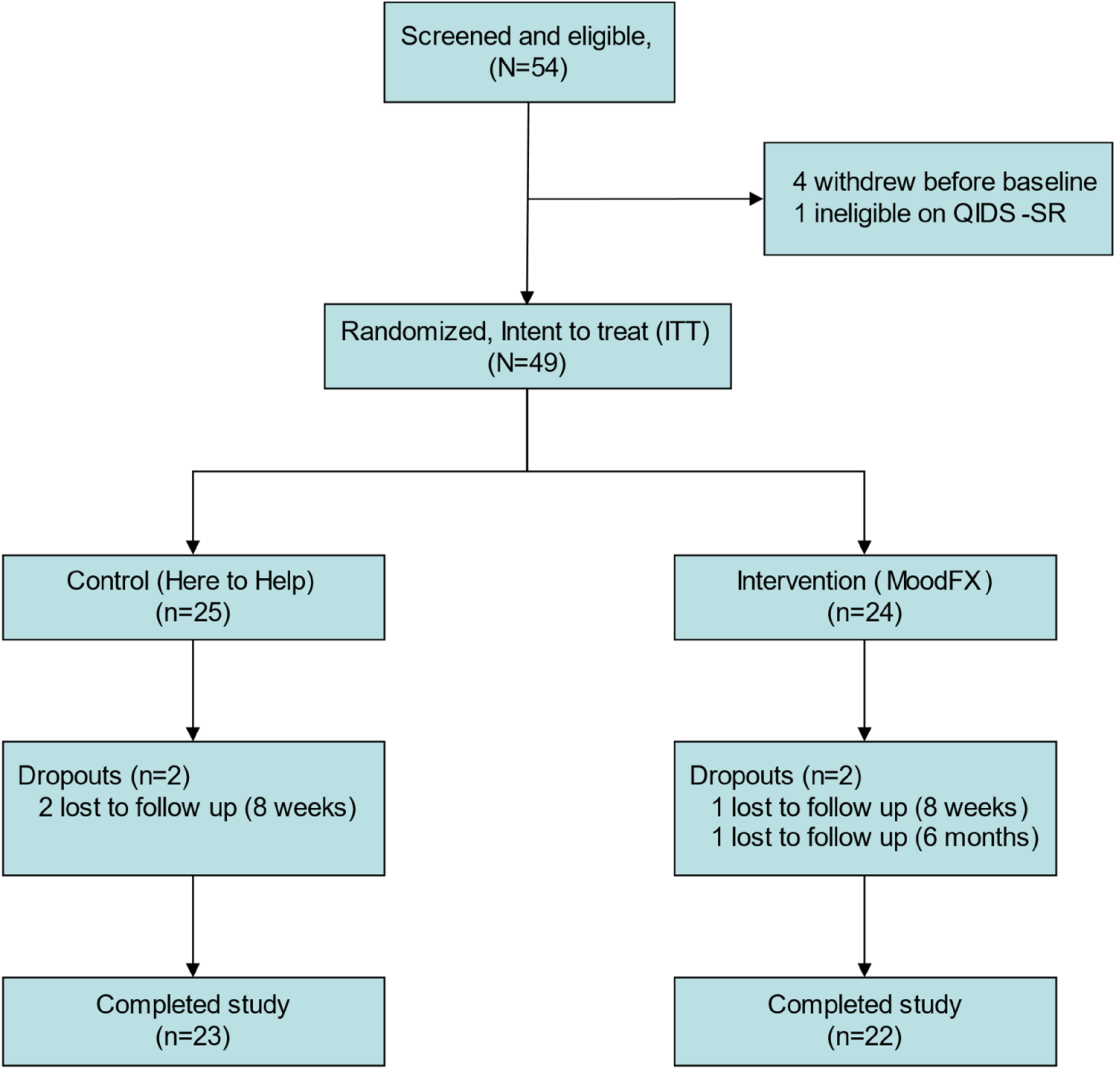
CONSORT diagram.

A total of 54 participants were recruited (Figure 1). Four participants withdrew before baseline assessment and one participant did not meet the QIDS-SR threshold criteria at baseline. The remaining 49 were randomized 1:1 to the intervention and control conditions, with 24 receiving MoodFX and 25 receiving the health information website. Table 2 lists demographic and clinical attributes for the sample, including age, sex, level of education, income, relationship status, computer usage frequency, primary diagnosis and others. There were no significant differences between the 2 conditions in any of these features. In regard to severity of depression, based on the baseline QIDS-SR scores, 25 of 49 (51%) participants were in the moderate category (QIDS-SR = 10–15), 21 of 49 (43%) were in the severe category (QIDS-SR = 16–20) and 3 of 49 (6%) were in the very severe category (QIDS-SR ≥ 21), with no differences in distribution between the 2 conditions. During the study, there were 4 participants that were lost to follow up (2 in each of the MoodFX and control conditions).

**Table 2.** Clinical and Demographic Information.

Item	Control (n = 25)	MoodFX (n = 24)	Total (N = 49)
Age, years (SD)	42.0 (13.3)	40.0 (14.6)	
Education, years (SD)	16.8 (2.5)	16.9 (2.1)	
Sex, F–M (%)	14 (56):11 (44)	15 (63):9 (38)	29 (59):20 (41)
Marital status			
• Married	• 12	• 14	• 26
• Single	• 3	• 4	• 7
• Other (separated, divorced, widowed)	• 10	• 6	• 16
Ethnicity			
• Asian	• 3	• 3	• 6
• Caucasian	• 13	• 13	• 26
• First nations	• 1	• 0	• 1
• Indo-Cdn	• 1	• 0	• 1
• African-Cdn	• 0	• 1	• 1
• Other	• 6	• 5	• 11
• No answer	• 1	• 2	• 3
Annual income			
• 0–40K	• 14	• 7	• 21
• 40–80K	• 6	• 9	• 15
• >80K	• 4	• 6	• 10
• No answer	• 1	• 2	• 3
Computer usage (%)			
• Frequent	• 22 (88)	• 18 (75)	• 40 (82)
• Some	• 3 (12)	• 5 (21)	• 8 (16)
• Never	• 0 (0)	• 1 (4)	• 1 (2)
Diagnosis (%)			
• Major depressive disorder	• 18 (72)	• 17 (71)	• 35 (71)
• Bipolar 2 disorder	• 4 (16)	• 7 (29)	• 11 (22)
• Bipolar 1 disorder	• 3 (12)	• 0 (0)	• 3 (6)

The MoodFX user survey was completed by 23 of 24 participants at the 6-month assessment. The primary usability outcome, the proportion of users who used MoodFX 3 or more times, was 18 of 23 (78%) (see Supplemental materials). An additional 4 participants (17%) reported that they used the app 2 times. One person indicated that they never used MoodFX at all. In terms of secondary usability outcomes, the SUS mean score was calculated to be 72.7 (SD 14.0). In terms of user sentiment, when summing responses of Strongly Agree and Agree, 11 of 23 (48%) felt that they would like to use the MoodFX app frequently, 20 of 23 (82%) felt very confident when using the app, 16 of 23 (70%) found the various functions well integrated, 18 of 23 (78%) felt that the app was overall easy to use and 21 of 23 (91%) believed that most people would be able to learn how to use it quickly. Only 3 of 23 (13%) of MoodFX users found the app unnecessarily complex, with 1 of 23 (4%) finding the tool very cumbersome to use.

**Figure 2.**
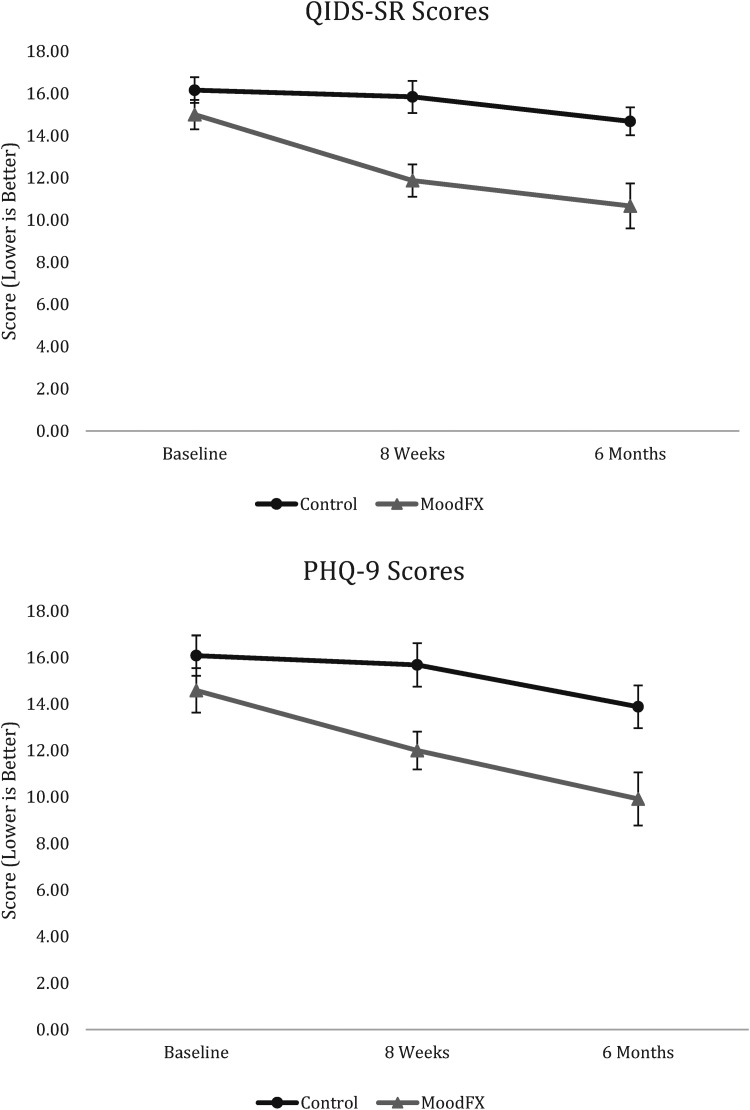
Mean QIDS-SR and PHQ-9 scores over time between control and MoodFX conditions. Error bars = SEM. Control n = 25. MoodFX n = 24. PHQ-9=Perceived Deficits Questionnaire, 9-item; QIDS-SR, Quick Inventory of Depressive Symptomatology, Self-Rated; SEM=standard error of the mean.

For the secondary clinical outcomes, mixed ANOVAs were conducted for test group * time. There was a significant interaction between condition and time on QIDS-SR scores (F_2,94 _= 3.981, *P* < 0.05), with significant and large differences between conditions at the 8-week (*P* = 0.001, d = 1.049, 95% confidence interval (CI) [0.445 to 1.642]) and 6-month time points (*P* = 0.002, d = 0.924, 95% CI [0.329 to 1.510]) (Figure 2). The effect sizes for the QIDS-SR at 8 weeks and 6 months were d = 1.05 and d = 0.92, respectively. For PHQ-9 scores, there was no significant interaction of condition * time (F_2,94 _= 2.196, *P* = 0.117), but there was a significant main effect of condition (F_1,47 _= 7.550, *P* < 0.01), as well as a significant main effect of time (F_2,94 _= 14.289, *P* < 0.001) (Figure 2). The effect sizes for the PHQ-9 at 8 weeks and 6 months were d = 0.85 and d = 0.78, respectively. For other secondary clinical measures, including anxiety symptoms, quality of life and functional measures, there were no statistically significant differences between conditions and there were greater proportions of missing data (Table 3 and Supplemental Table S1).

**Table 3.** Main Secondary Clinical Outcomes.

		Control Condition			MoodFX		
		Mean	SD	n	Mean	SD	n
QIDS-SR*	Baseline	16.16	3.05	25	15.00	3.43	24
	Week 8	15.84	3.80	25	11.88	3.76	24
	Month 6	14.68	3.30	25	10.67	5.21	24
PHQ-9*	Baseline	16.08	4.36	25	14.58	4.67	24
	Week 8	15.68	4.69	25	12.00	3.98	24
	Month 6	13.88	4.58	25	9.92	5.62	24
GAD-7	Baseline	8.72	5.67	25	8.83	6.25	24
	Week 8	8.12	5.71	25	9.26	5.52	23
	Month 6	7.29	6.05	24	8.91	5.53	23
SDS Average Score	Baseline	7.23	3.20	25	6.86	2.73	24
	Week 8	6.45	3.07	24	6.84	2.32	22
	Month 6	5.94	2.69	21	6.72	2.82	22
LEAPS Productivity Subscale	Baseline	3.50	2.97	12	4.44	2.56	16
	Week 8	2.42	1.83	12	3.40	2.56	15
	Month 6	2.80	2.94	10	3.46	2.82	13
QLESQ-SF Total	Baseline	0.41	0.20	25	0.42	0.16	24
	Week 8	0.42	0.18	24	0.47	0.14	22
	Month 6	0.46	0.15	21	0.45	0.23	22

* Significant differences between conditions, by mixed analysis of variance, see text.

GAD-7=Generalized Anxiety Disorder scale; LEAPS=Lam Employment Absence and Productivity Scale; PHQ-9=Personal Health Questionnaire 9-item; QIDS-SR=Quick Inventory of Depressive Symptomatology, Self-Rated; QLESQ-SF=Quality of Life, Enjoyment and Satisfaction Questionnaire, Short Form; SDS=Sheehan Disability Scale.

In terms of response rates (50% or greater improvement from baseline), at 8 weeks the MoodFX response rate for the QIDS-SR was higher than the control group, but narrowly missed statistical significance (4 of 24 [17%] vs. 0 of 25 [0%], respectively, *P* = 0.050 by Fisher's exact test), whereas at 6 months the MoodFX response rate was significantly higher (7 of 24 [29%] vs. 0 of 25 [0%], *P* = 0.004). Similarly, the 8-week PHQ-9 response rate was not significantly different between the 2 groups (3 of 24 [13%] vs. 1 of 25 [4%], *P* = 0.34), but the 6-month PHQ-9 response rate was significantly higher for the MoodFX group (10 of 24 [42%] vs. 2 of 25 [8%], *P* = 0.008). The percentage of participants no longer meeting the entry QIDS-SR severity criterion (i.e., those with QIDS-SR < 10) was significantly higher in the MoodFX group compared to the control condition (at 8 weeks, 6 of 24 [25%] vs. 1 of 25 [4%], respectively, *P* = 0.049 by Fisher's exact test; at 6 months, 12 of 24 [50%] vs. 1 of 25 [4%], respectively, *P* < 0.001).

## Discussion

In this pilot study, we investigated the usability of MoodFX as a web-based platform optimized for mobile devices to facilitate outcome measurement for depression. The majority (78%) of participants used MoodFX 3 times or more over the past 6 months, a minimally adequate usage frequency for MBC, with a further 17% using it twice. Moreover, most patients found the web app easy to use, with an average score of 72.7 on the SUS, corresponding to 65th–69th percentile^21^ and a “good” rating.^22^ Compared to other symptom tracking apps,^23–26^ MoodFX has more questions and takes longer to complete. However, its high usability score, user feedback and engagement metrics suggest that the length was not a significant drawback, with the caveat that comparisons between studies are difficult due to widely varying populations and criteria for adherence.^27^

The secondary clinical outcomes showed that self-rated depression symptom scores, as measured by the QIDS-SR, were significantly improved when participants had access to MoodFX compared to the control condition, with large effect sizes at 8-week and 6-month follow up (d = 1.05 and d = 0.92, respectively). There was no significant improvement on PHQ-9 scores over time (*P* = 0.11), but the effect sizes at 8-week and 6-month follow up were also large (d = 0.85 and d = 0.78, respectively), driven by significant main effects of time and condition; both groups improved over time, and the group assigned to MoodFX had better PHQ-9 scores overall compared to the control condition. Furthermore, significantly more patients in the MoodFX group versus the control condition demonstrated response on the QIDS-SR (29% vs. 0%) and the PHQ-9 (42% vs. 8%) at 6 months, and no longer met the threshold QIDS-SR entry criterion (50% vs. 4%). The differences in significance patterns between the QIDS-SR and PHQ-9 may be related to type II errors because of the small sample size. This is also possible for the results of other measures such as anxiety, quality of life and functioning scores, which were not significantly different between the 2 conditions. For anxiety, the baseline GAD-7 scores were also lower than the depressive symptom measures, hence, there is less likelihood of improvement with an intervention focused on depression. Since recovery of functioning and quality of life may lag behind recovery in symptoms,^28,29^ it is possible that these were not well captured during our study duration. Some of the secondary clinical measures also had higher rates of missing data which reduced power to detect a difference between groups.

As a user-centric mood tracking app, MoodFX addresses some of the barriers for outcome measurement in clinical practice, such as curbing the cost, resource, and time burden on clinicians, and making it more convenient to administer rating scales. Furthermore, using a web-based application prevents reliance on synchronous interactions with the clinicians, which may take place on an irregular basis or, in certain extenuating circumstances such as the COVID-19 pandemic, may not take place at all. The online tool can be used at the convenience of the patient, promoting use on a more frequent basis. This also allows for more accurate monitoring of symptoms, as individuals do not have to wait until an in-person visit to complete the scales, during which they may be limited in their memory of events that took place, or their general mood and functioning.^30^ Measurements on the patients’ own time may also be more sensitive for symptoms and suicidality compared to the same paper-and-pencil scales in a clinical setting.^26^ However, in this study we do not know whether patients used MoodFX together with their treating physicians, or if they used it on their own. Thus, it remains unclear if any potential benefits are due to improving clinical care with MBC or by facilitating self-management of depression. In addition, using a monitoring app without guidance may carry potential harm, such as by discouraging users with scores that fail to improve over time. In MoodFX, these risks are mitigated by the FAQ section that explains how scores are used and why they may not improve.

Digital healthcare tools can also risk deepening the digital divide, whereby unequal access or literacy with digital resources can result in unequal health outcomes.^31^ While access to the internet is almost ubiquitous in Canada and elsewhere, technological literacy may be more of an issue. Since our study participants, on average, were highly educated and familiar with computers, our results may not translate to all populations. The lack of languages other than French and English at this time also could be an access barrier to ethnocultural minorities.^32^ The familiarity and comfort of clinicians with digital tools may also impact the utility of a resource such as MoodFX. Implementing MoodFX as a smartphone app instead of a website in its next iteration could foreseeably streamline the user experience and reduce the barrier for ongoing use, such as by delivering reminders with notifications rather than with emails or SMS.

There are also several limitations to our study. First, this was a pilot study with a small sample size that limited power to detect differences in the secondary measures. Second, the intention of the study was primarily to assess usability, so factors such as how participants or clinicians applied the MoodFX results were not standardized or assessed at all. This limits conclusions that can be drawn about the improvements in the secondary clinical outcomes. Third, back-end user data were not available, so it was not possible to track MoodFX usage patterns, which would be more informative than the self-reported usage. Fifth, we do not know whether participants in the control condition used rating scales; however, if they did, this would bias against finding differences in outcomes and lead to smaller effect sizes. Future studies should address these limitations to better understand how e-Health tools are used to affect outcomes.

In conclusion, our results from this pilot study support the usability of MoodFX for outcome measurement during depression care and provide preliminary support for its association with reducing depressive symptoms. Further studies should focus on patient outcomes with larger sample sizes, collect back-end usage data, and assess how MoodFX is used by both patients and clinicians to better understand and situate its role in measurement-based patient care.

## Supplemental Material

sj-docx-1-cpa-10.1177_07067437241245331 - Supplemental material for A Randomized Evaluation of MoodFX, a Patient-Centred e-Health Tool to Support Outcome Measurement for Depression: Une évaluation randomisée de MoodFX, un outil de santé en ligne centré sur le patient pour soutenir la mesure du résultat dans la dépression. Supplemental material, sj-docx-1-cpa-10.1177_07067437241245331 for A Randomized Evaluation of MoodFX, a Patient-Centred e-Health Tool to Support Outcome Measurement for Depression: Une évaluation randomisée de MoodFX, un outil de santé en ligne centré sur le patient pour soutenir la mesure du résultat dans la dépression. by Victor W. Li, Jaspreet Sahota, Deea K. Dev, Dib D. Gill, Vanessa C. Evans, Auby Axler, Trisha Chakrabarty, André Do, Kamyar Keramatian, John-Jose Nunez, Edwin M. Tam, Lakshmi N. Yatham, Erin E. Michalak, Jill K. Murphy and Raymond W. Lam in The Canadian Journal of Psychiatry
