# Supplementary material for: A Randomized Evaluation of MoodFX, a Patient-Centred e-Health Tool to Support Outcome Measurement for Depression: Une évaluation randomisée de MoodFX, un outil de santé en ligne centré sur le patient pour soutenir la mesure du résultat dans la dépression
Source: Can J Psychiatry. 2024 Apr 11;69(7):493–502. doi: 10.1177/07067437241245331 (PMC11168344; doi:10.1177/07067437241245331)
Supplement: sj-docx-1-cpa-10.1177_07067437241245331 - Supplemental material for A Randomized Evaluation of MoodFX, a Patient-Centred e-Health Tool to Support Outcome Measurement for Depression: Une évaluation randomisée de MoodFX, un outil de santé en ligne centré sur le patient pour soutenir la mesure du résul [file sj-docx-1-cpa-10.1177_07067437241245331.docx]

**Supplementary Materials**

User Survey Results (n=23), final, Jan-2-2023.

| usersurvey_# | Question | Score | Rating | N | % | Merged % |
| --- | --- | --- | --- | --- | --- | --- |
| 1 | I think that I would like to use the tool frequently. | 4 | Strongly agree | 1 | 4.3 | 47.8 |
|  |  | 3 | Agree | 10 | 43.5 |  |
|  |  | 2 | Neutral | 5 | 21.7 | 21.7 |
|  |  | 1 | Disagree | 7 | 30.4 | 30.4 |
|  | Average 2.125 -> 1.125 | 0 | Strongly disagree | 0 | 0 |  |

| usersurvey_# | Question | Score | Rating | N | % | Merged % |
| --- | --- | --- | --- | --- | --- | --- |
| 2 | I found the tool unnecessarily complex. | 0 | Strongly agree | 0 | 0 | 13.0 |
|  |  | 1 | Agree | 3 | 13.0 |  |
|  |  | 2 | Neutral | 2 | 8.7 | 65.2 |
|  |  | 3 | Disagree | 15 | 65.2 | 13.0 |
|  | 1.167-> 3.83 | 4 | Strongly disagree | 3 | 13.0 |  |

| usersurvey_# | Question | Score | Rating | N | % | Merged % |
| --- | --- | --- | --- | --- | --- | --- |
| 3 | I thought the tool was easy to use. | 4 | Strongly agree | 3 | 13.0 | 78.2 |
|  |  | 3 | Agree | 15 | 65.2 |  |
|  |  | 2 | Neutral | 3 | 13.0 | 13.0 |
|  |  | 1 | Disagree | 2 | 8.7 | 8.7 |
|  | 2.71->1.71 | 0 | Strongly disagree | 0 | 0 |  |

| usersurvey_# | Question | Score | Rating | N | % | Merged % |
| --- | --- | --- | --- | --- | --- | --- |
| 4 | I think that I would need the support of a technical person to be able to use the tool. | 0 | Strongly agree | 0 | 0 | 8.7 |
|  |  | 1 | Agree | 2 | 8.7 |  |
|  |  | 2 | Neutral | 0 | 0 | 0 |
|  |  | 3 | Disagree | 6 | 26.1 | 91.3 |
|  | 0.5->4.5 | 4 | Strongly disagree | 15 | 65.2 |  |

| usersurvey_# | Question | Score | Rating | N | % | Merged % |
| --- | --- | --- | --- | --- | --- | --- |
| 5 | I found the various functions in the tool were well integrated. | 4 | Strongly agree | 3 | 13.0 | 69.5 |
|  |  | 3 | Agree | 13 | 56.5 |  |
|  |  | 2 | Neutral | 6 | 26.1 | 26.1 |
|  |  | 1 | Disagree | 1 | 4.3 | 4.3 |
|  | 2.667->1.667 | 0 | Strongly disagree | 0 | 0 |  |
| usersurvey_# | Question | Score | Rating | N | % | Merged % |
| 6 | I thought there was too much inconsistency in the tool. | 0 | Strongly agree | 0 | 0 | 0 |
|  |  | 1 | Agree | 0 | 0 |  |
|  |  | 2 | Neutral | 5 | 21.7 | 21.7 |
|  |  | 3 | Disagree | 10 | 43.5 | 78.3 |
|  | 0.833->4.167 | 4 | Strongly disagree | 8 | 34.8 |  |

| usersurvey_# | Question | Score | Rating | N | % | Merged % |
| --- | --- | --- | --- | --- | --- | --- |
| 7 | I would imagine that most people would learn to use the tool very quickly. | 4 | Strongly agree | 4 | 17.4 | 91.3 |
|  |  | 3 | Agree | 17 | 73.9 |  |
|  |  | 2 | Neutral | 1 | 4.3 | 4.3 |
|  |  | 1 | Disagree | 1 | 4.3 | 4.3 |
|  | 2.917->1.917 | 0 | Strongly disagree | 0 | 0 |  |

| usersurvey_# | Question | Score | Rating | N | % | Merged % |
| --- | --- | --- | --- | --- | --- | --- |
| 8 | I found the tool very cumbersome to use. | 0 | Strongly agree | 1 | 4.3 | 4.3 |
|  |  | 1 | Agree | 0 | 0 |  |
|  |  | 2 | Neutral | 5 | 21.7 | 21.7 |
|  |  | 3 | Disagree | 15 | 65.2 | 73.9 |
|  | 1.208->3.792 | 4 | Strongly disagree | 2 | 8.7 |  |

| usersurvey_# | Question | Score | Rating | N | % | Merged % |
| --- | --- | --- | --- | --- | --- | --- |
| 9 | I felt very confident using the tool. | 4 | Strongly agree | 2 | 8.7 | 82.0 |
|  |  | 3 | Agree | 18 | 78.3 |  |
|  |  | 2 | Neutral | 1 | 4.3 | 4.3 |
|  |  | 1 | Disagree | 2 | 8.7 | 8.7 |
|  | 2.75->1.75 | 0 | Strongly disagree | 0 | 0 |  |

| usersurvey_# | Question | Score | Rating | N | % | Merged % |
| --- | --- | --- | --- | --- | --- | --- |
| 10 | I needed to learn a lot of things before I could get going with the tool. | 0 | Strongly agree | 0 | 0 | 8.7 |
|  |  | 1 | Agree | 2 | 8.7 |  |
|  |  | 2 | Neutral | 1 | 4.3 | 4.3 |
|  |  | 3 | Disagree | 10 | 43.5 | 87.0 |
|  | 0.75->4.25 | 4 | Strongly disagree | 10 | 43.5 |  |

| usersurvey_# | Question | Score | Rating | N | % |
| --- | --- | --- | --- | --- | --- |
| 11 | Over the last six months, how many times did you use the tool? | 0 | Never | 1 | 4.3 |
|  |  | 1 | Once | 0 | 0 |
|  |  | 2 | Twice | 4 | 17.4 |
|  |  | 3 | Three or more times | 18 | 78.3 |

**Supplementary Table S1: Other Secondary Clinical Outcomes*.**

|  |  | Control | | | MoodFX | | |
| --- | --- | --- | --- | --- | --- | --- | --- |
|  |  | Mean | SD | n | Mean | SD | n |
| PDQ-5 | Baseline | 10.17 | 5.67 | 25 | 11.84 | 5.49 | 24 |
|  | Week 8 | 10.45 | 5.26 | 24 | 10.46 | 5.12 | 22 |
|  | Month 6 | 9.64 | 5.78 | 21 | 10.95 | 4.98 | 22 |
| LEAPS Total Score | Baseline | 10.06 | 5.74 | 12 | 8.67 | 5.63 | 16 |
|  | Week 8 | 9.80 | 5.58 | 12 | 7.17 | 3.69 | 15 |
|  | Month 6 | 8.85 | 5.96 | 10 | 6.60 | 5.64 | 13 |
| QLESQ-SF Medication Satisfaction | Baseline | 2.52 | 0.98 | 24 | 3.54 | 0.88 | 21 |
|  | Week 8 | 3.00 | 0.91 | 20 | 3.45 | 0.69 | 18 |
|  | Month 6 | 3.50 | 1.10 | 17 | 3.24 | 0.56 | 18 |
| QLESQ-SF Overall Satisfaction | Baseline | 2.46 | 0.98 | 25 | 2.40 | 1.08 | 24 |
|  | Week 8 | 2.73 | 1.12 | 24 | 2.67 | 0.96 | 22 |
|  | Month 6 | 2.77 | 1.15 | 21 | 3.05 | 0.86 | 22 |
| EQ-5D-5L | Baseline | 10.50 | 2.87 | 25 | 11.24 | 4.13 | 24 |
|  | Week 8 | 10.41 | 2.79 | 24 | 10.79 | 3.61 | 22 |
|  | Month 6 | 9.73 | 3.17 | 21 | 10.67 | 3.25 | 22 |
| EQ-5D-5L VAS | Baseline | 50.92 | 18.22 | 25 | 51.28 | 20.42 | 24 |
|  | Week 8 | 55.64 | 16.93 | 24 | 54.29 | 19.85 | 22 |
|  | Month 6 | 58.95 | 20.42 | 21 | 53.00 | 22.80 | 22 |
| FIBSER | Baseline | 1.63 | 1.74 | 25 | 1.60 | 1.83 | 24 |
|  | Week 8 | 1.32 | 1.36 | 24 | 1.08 | 1.47 | 22 |
|  | Month 6 | 1.09 | 1.31 | 21 | 1.10 | 1.30 | 22 |

No significant differences between conditions for any of the outcomes.

EQ-5D-5L, EuroQol Group: 5 Dimension, 5 Level scale; FIBSER, Frequency, Intensity, and Burden of Side Effects Ratings; LEAPS, Lam Employment Absence and Productivity Scale; PHQ-9, Personal Health Questionnaire 9-item; PDQ-5, Perceived Deficits Questionnaire, 5-item; QIDS-SR, Quick Inventory of Depressive Symptomatology, Self-Rated; QLESQ-SF, Quality of Life, Enjoyment and Satisfaction Questionnaire, Short Form; SDS, Sheehan Disability Scale; VAS, Visual Analog Scale.
